# Transcriptional response of *Wolbachia*-transinfected *Aedes aegypti* mosquito cells to dengue virus at early stages of infection

**DOI:** 10.1099/jgv.0.001694

**Published:** 2022-01-10

**Authors:** Michael Leitner, Kayvan Etebari, Sassan Asgari

**Affiliations:** ^1^​ Australian Infectious Disease Research Centre, School of Biological Sciences, The University of Queensland, Brisbane, Australia

**Keywords:** *Aedes aegypti*, dengue virus, differential gene expression, transcriptome, *Wolbachia*

## Abstract

Mosquito-borne flaviviruses are responsible for viral infections and represent a considerable public health burden. *Aedes aegypti* is the principal vector of dengue virus (DENV), therefore understanding the intrinsic virus–host interactions is vital, particularly in the presence of the endosymbiont *Wolbachia,* which blocks virus replication in mosquitoes. Here, we examined the transcriptional response of *Wolbachia *-transinfected *Ae. aegypti* Aag2 cells to DENV infection. We identified differentially expressed immune genes that play a key role in the activation of anti-viral defence such as the Toll and immune deficiency pathways. Further, genes encoding cytosine and N^6^-adenosine methyltransferases and SUMOylation, involved in post-transcriptional modifications, an antioxidant enzyme, and heat-shock response were up-regulated at the early stages of DENV infection and are reported here for the first time. Additionally, several long non-coding RNAs were among the differentially regulated genes. Our results provide insight into *Wolbachia *-transinfected *Ae. aegypti*’s initial virus recognition and transcriptional response to DENV infection.

Dengue virus (DENV) is a mosquito-borne human pathogen responsible for an estimated 390 million dengue infections per year [1, 2]. DENV belongs to the *Flaviviridae* family consisting of four serotypes (DENV-1 to −4), and has a single-stranded positive-sense RNA genome that encodes three structural and seven non-structural proteins [2]. The virus is primarily transmitted to humans by the bites of female mosquitoes of the genus *Aedes*, especially *Aedes aegypti* [3, 4]. Mosquitoes such as *Ae. aegypti* lack an adaptive immune system depending entirely on their innate immune system to fight and control arbovirus replication [5–8]. Presently, there is only one vaccine with limited use in seropositive patients naturally infected with DENV and no effective antiviral therapies available, meaning effective transmission control alternatives to prevent the spread of DENV are being investigated [9, 10]. One successful attempt has been the transinfection of the endosymbiotic bacterium *Wolbachia pipientis* into *Ae. aegypti* mosquitoes as a biocontrol approach. It has been shown that *Wolbachia* inhibits viral replication and dissemination of several arboviruses in transinfected *Ae. aegypti* and reduces virus transmission to humans [11–15]. Nonetheless, the mutually beneficial symbiotic relationship and *Wolbachia *-mediated viral inhibition are not yet fully understood.

The transcriptional response of *Ae. aegypti* to viral infections has been extensively investigated, however, to our knowledge, exclusively at later stages of infection considering time points of 1–14 days post-infection (dpi) and in the absence of *Wolbachia* [16–20]. Here, we examined the transcriptional response of *Ae. aegypti* to DENV infection in *Wolbachia *-transinfected *Ae. aegypti* Aag2 cells at early time points after infection to identify host genes that respond to infection. Early time points were chosen as previous studies have indicated that viruses belonging to *Togaviridae* and *Flaviviridae* families of RNA viruses, including DENV-2, initiate replication from around 6 hpi in mosquito cells [21, 22], and that the anti-viral response exerted by *Wolbachia* occurs at very early hours post-infection [22, 23]. For this, we compared the transcriptional response of *Ae. aegypti* to DENV infection in a *Wolbachia w*AlbB strain-transinfected Aag2-derived cell line, using RNA sequencing (RNA-Seq) data recently generated to compare transcriptional response of *Wolbachia w*AlbB strain to DENV infection [24]. This analysis also provides complementary information to the previous study [24] by analysing host cell responses to DENV infection in the same setting. In the study, Aag2.*w*AlbB cells infected with DENV serotype 2 (DENV-2) East Timor strain (ET-300) at the multiplicity of infection (MOI) of 1 were used, with virus-infected and uninfected cells collected at 1, 6, and 24 h post-infection (hpi) in three biological replicates for each time point. In our experimental design, we used matched time points for virus-infected and uninfected samples to take into consideration possible changes of gene expression over time. The density of *w*AlbB *Wolbachia* was about 120 per cell determined at the time of setting up the experiment for RNA-Seq [24]. *Wolbachia* density was not further monitored throughout the experiment as we assumed there would not be significant changes over 24 h. Further, using matched time points for infected and uninfected samples should consider the potential changes in *Wolbachia* density over the 24 h period.

Sample and RNA preparation, library construction, and RNA-Seq have been described previously [24], with the RNA-Seq data available on Sequence Read Archive (SRA) under the Project ID PRJNA669319. RNA-Seq data analysis was carried out using CLC Genomics Workbench v20.0.2 (CLC-GWB, Qiagen) as described previously [24]. Briefly, raw paired-end reads were adapter-trimmed and low-quality sequences removed (quality score=0.05) using the trim reads sequence tool in CLC-GWB. The high-quality trimmed paired-end reads were mapped to a combined reference genome assembly containing the *Ae. aegypti* AaegL5.0 chromosome 1–3 (NC_035107.1, NC_035108.1 and NC_035109.1) and mitogenome (MF194022.1), *Wolbachia w*AlbB genome (CP031221.1), Cell fusing agent virus (CFAV; NC_001564.2), *Aedes albopictus* negev-like virus (AaNLV; MK879802.1), and DENV-2 ET-300 genome (MT921572.1). Aag2.*w*AlbB cells are persistently infected with CFAV and AalNLV [25]. The mapping setting ‘Genome annotated with genes and transcripts suitable for Eukaryotes’ was used, which considers splicing. Mapping parameters were minimum length fraction of 0.8, minimum similarity fraction of 0.9, and maximum number of hits for a read of 30 to capture multimapping repetitive elements. After adapter trimming and quality filtering, in total, 1 629 758 722 high quality reads with 150 bp paired-ends were generated from DENV-2 infected and uninfected Aag2.*w*AlbB cells.

To identify differentially expressed genes (DEGs) between DENV-infected and uninfected Aag2.*w*AlbB samples, the CLC-GWB differential expression for RNA-Seq tool with the default parameters was used. To calculate effective library sizes, Trimmed Mean of M values (TMM) normalisation was used [26]. The CLC-GWB differential expression for RNA-Seq tool uses multi-factorial statistics based on a negative binomial generalised linear model (GLM) [27]. Differential gene expression between DENV-infected and uninfected samples was evaluated at 1, 6, and 24 hpi using ‘All group pairs’ analysis in CLC-GWB, which tests for differences between all pairs of groups in a factor, including a Benjamini-Hochberg correction for multiple comparisons, and adjusts *P* values. Significance of DEGs was assessed using the Wald test and results were then filtered based on a fold-change of ≥2.0, and false discovery rate (FDR) *P* value of <0.05.

When DENV-infected and uninfected samples at each time point were compared, a total of 2317 genes were significantly differentially expressed across the three time points combined, considering fold-change of ≥2.0 and an FDR *P* value of <0.05; 590, 1196, and 531 DEGs at 1, 6, and 24 hpi, respectively (Fig. 1a, Table S1, available in the online version of this article). Specifically, 342 (58.0 %), 966 (80.8 %), and 378 (71.2 %) were up-regulated at 1, 6, and 24 hpi in contrast to 248 (42.0 %), 230 (19.2 %), and 153 (28.8 %) down-regulated genes at the three time points, respectively (Fig. 1a). There was a total of 453 overlapping DEGs between the time points (Fig. 1c, Table S1). More precisely, 82 overlapping DEGs were consistently expressed across all three time points, with 62 (75.6 %) up-regulated, and 20 (24.5 %) down-regulated (Fig. 1c, Table S1). When comparing 1 vs 6, 1 vs 24, and 6 vs 24 hpi, a total of 122, 26, and 223 DEGs overlapped between these time points, respectively (Fig. 1c, Table S1). Specifically, 89 (73.0 %), 12 (46.2 %), and 204 (91.5 %) were up-regulated in contrast to 33 (27.0 %), 14 (53.8 %), and 19 (8.5 %) down-regulated genes between these time points, respectively (Fig. 1c, Table S1). Further, a total of 258 long non-coding RNAs (lncRNAs) were significantly differentially expressed across the three time points combined; 90, 115, 53 lncRNAs at 1, 6, and 24 hpi, respectively (Fig. 1b, Table S1). In particular, 27 (30.0 %), 52 (45.2 %), and 24 (45.3 %) were up-regulated at 1, 6, and 24 hpi compared to 63 (70.0 %), 63 (54.8 %), and 29 (54.7 %) down-regulated genes at the three time points, respectively (Fig. 1b, Table S2).

**Fig. 1.**
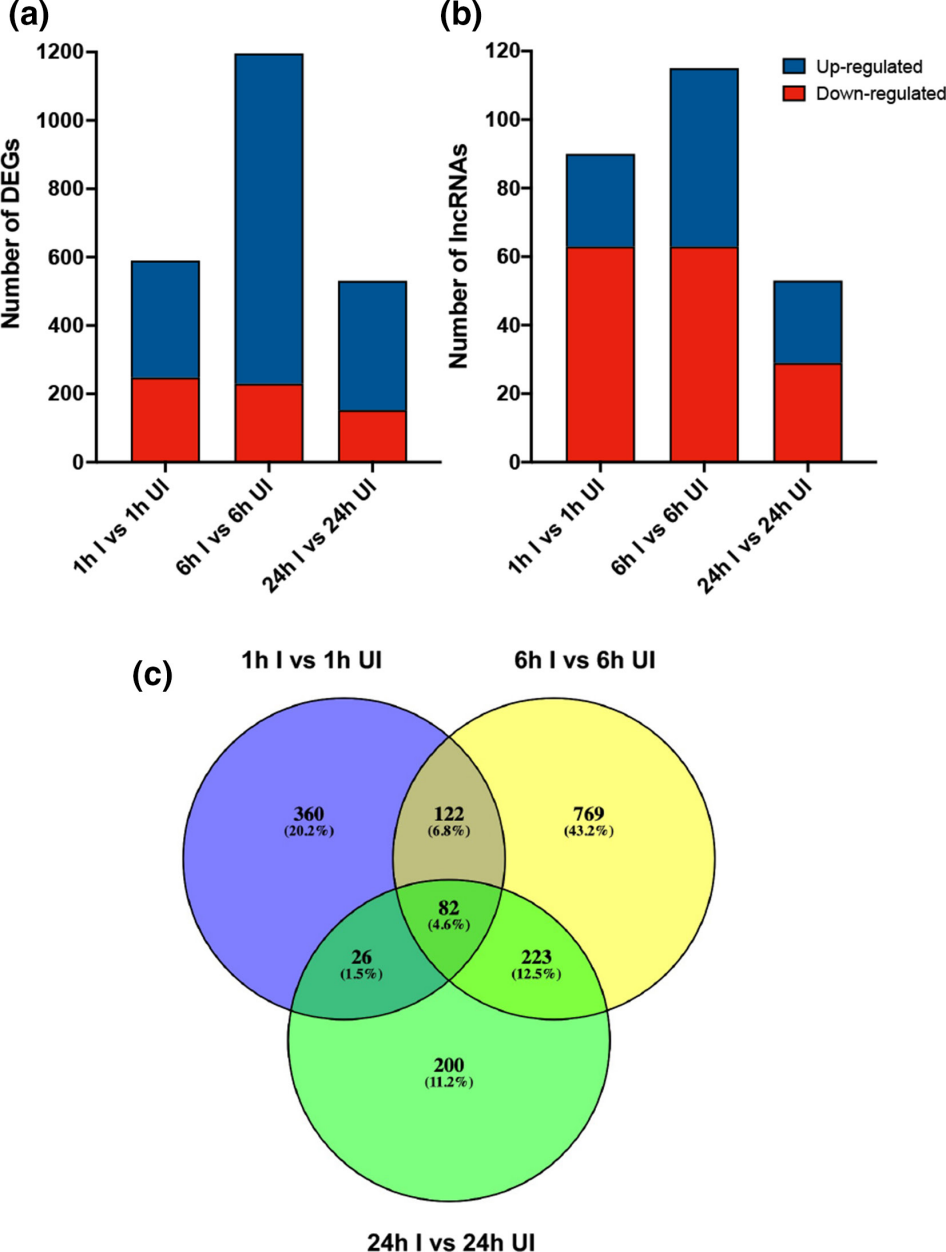
Differentially expressed protein-coding genes (DEGs) and long non-coding RNAs (lncRNAs) in response to DENV infection in *Wolbachia *-transinfected Aag2 cells. The bars represent the number of (**a**) DEGs and (**b**) lncRNAs identified using a fold-change of ≥2.0 and an adjusted *P* value of <0.05. The up-regulated DEGs and lncRNAs are shown in blue and the down-regulated DEGs and lncRNAs are shown in red. (**c**) Venn diagram showing DEGs in DENV-infected versus uninfected *Wolbachia *-transinfected Aag2 cells. A total of 453 overlapping DEGs at more than one time point were found. Each coloured circle represents a sample collection time point.

A representative selection of differentially expressed *Ae. aegypti* genes identified by RNA-Seq analysis were validated by RT-qPCR. NCBI Primer-blast primer design tool [28] was used to design primers for the selected DEGs. The primers are listed in Table S3. New RNA samples were generated by repeating the infection experiment with DENV-2 ET-300 under identical conditions as before by collecting infected and uninfected Aag2.*w*AlbB cells at 1, 6, and 24 hpi. RNA extraction, reverse transcription and qPCR were performed as described previously [24]. *Ae. aegypti* ribosomal protein subunit 17 gene (RPS17) was used as the normalizing gene [15]. The relative abundance of RPS17 and selected DEGs was determined using the relative quantification method as described previously [29]. The results showed an overall consistency between RNA-Seq and RT-qPCR, when DEGs in DENV-infected Aag2.*w*AlbB cells in respect to DENV infection were considered (Fig. 2). While *Wolbachia* density was determined to be at about 120 per cell for the RNA-Seq experiment, for the RT-qPCR experiment there was a drop to 75 *Wolbachia* per cell. Nevertheless, RNA-Seq and RT-qPCR validation experiments showed an overall consistency despite the difference in *Wolbachia* density. The expression of the first four genes shown in Fig. 2 were also assessed in uninfected Aag2.*w*AlbB cells, which showed consistency with RNA-Seq data (Fig. S1).

**Fig. 2.**
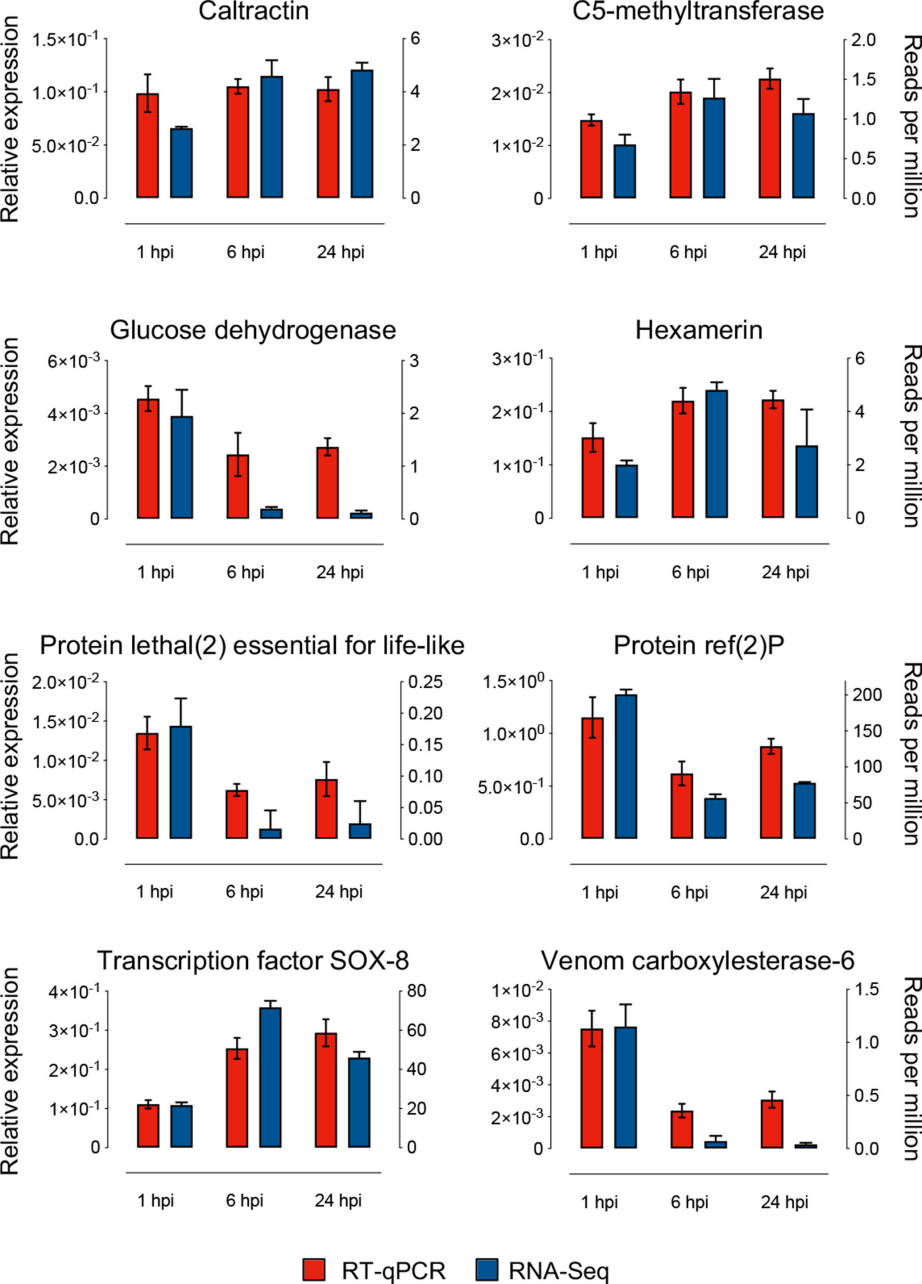
RT-qPCR validation of the differentially expressed genes (DEGs). The bar graphs represent the RNA-Seq normalized gene reads as counts per million and RT-qPCR relative expression results of the DEGs in DENV-infected *Wolbachia *-transinfected Aag2 cells at time points 1, 6, and 24 hpi. The error bars represent mean normalized expression (MNE) from the three biological replicates.

All the differentially expressed genes were uploaded to the Blast2GO [30] server for functional annotation and Gene Ontology (GO) analysis. We used blast, InterProScan [31], enzyme classification codes (EC) and EggNOG [32] to reveal the GO terms of the differentially expressed sequences. An enrichment analysis using Fisher’s Exact Test was done using all AaegL5.0 annotated genes as the reference dataset by FatiGO package, which is integrated in Blast2GO. Overrepresented terms were considered if the *P* value was lower than 0.05 and their enrichment fold change was greater than two. A bar chart was produced showing the *P* values and fold changes for the top 20 most abundant GO terms for each category of biological process, molecular function, and cellular components at each time point (Fig. 3). Most enriched DEGs were related to defence response to bacterium, structural constituent of ribosome, and extracellular space at 1 hpi; cellular amide metabolic process, structural constituent of ribosome, and membrane-enclosed lumen at 6 hpi; and organonitrogen compound biosynthetic process, oxidoreductase activity, and membrane-enclosed lumen at 24 hpi for GO terms of biological process, molecular function, and cellular compartment, respectively (Fig. 3). When comparing the 20 most abundant GO term categories to each other there was little overlap between all three time points, zero, two (structural constituent of ribosome and disulfide oxidoreductase activity), and three (ribosome, ribosomal subunit, and mitochondrial protein-containing complex) of most abundant GO terms based on biological process, molecular function, and cellular component, respectively (Fig. 3).

**Fig. 3.**
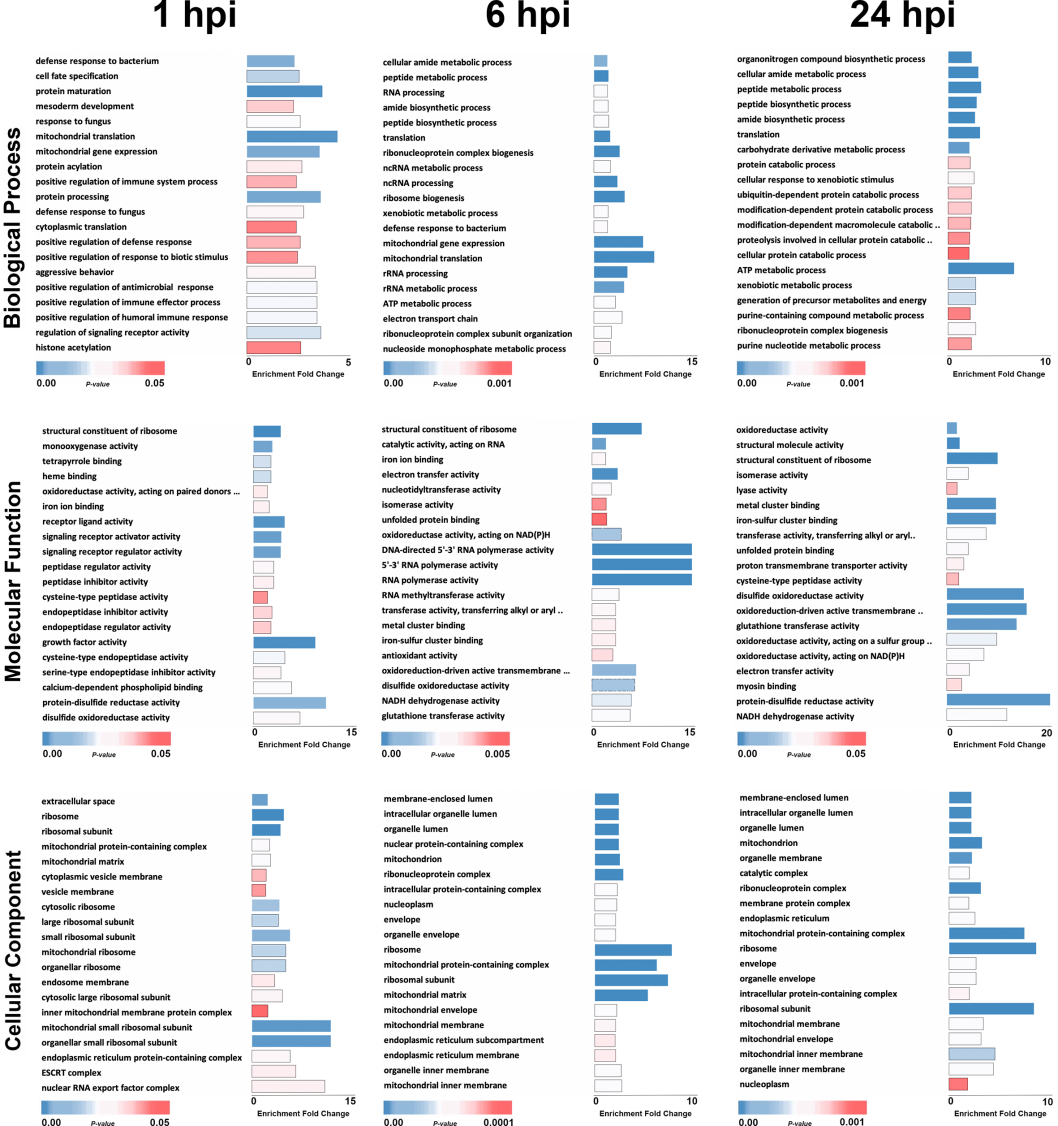
Gene Ontology (GO) analysis of differentially expressed genes (DEGs) in response to DENV infection in *Wolbachia *-transinfected Aag2 cells. The bar graphs represent the top 20 most abundant gene ontology terms for each category of biological process, molecular function, and cellular components at time points 1, 6, and 24 hpi. The x-axis shows the fold change enrichment and significance of each GO term, and the y-axis the GO term category names.

Of the 62 up-regulated overlapping DEGs almost all remained up-regulated across all the three time points, except for one, a START domain-containing protein 10, which was up-regulated at 1, but down-regulated at 6, and 24 hpi (Table S1). However, the 20 down-regulated overlapping DEGs remained down-regulated across all the three time points (Table S1).

Genes encoding essential cellular, ribosomal, nuclear, developmental, metabolic, transport/trafficking, stress response functions, and mitochondrial proteins were significantly up-regulated due to DENV infection. Notably, a peroxiredoxin-6 (prx-6), an apoptosis regulator (BAX) inhibitor, a dolichol-phosphate mannosyltransferase subunit 3 (DPM3), and two lethal(2)essential for life (l(2)efl) proteins were among the persistently up-regulated genes.

Peroxiredoxins are antioxidant enzymes part of the thioredoxin system that detoxify reactive oxygen species (ROS), cytotoxic peroxide, and reactive nitrogen oxide species (RNOS) protecting proteins and cells against oxidative damage [33–36]. Viral infections, including that of DENV, can induce oxidative stress in infected mosquito cells, however, through the activation of antioxidant enzymes cells protect themselves against cellular damage and virus infection [36–38]. Recent studies examining the transcriptional response of *Ae. aegypti* mosquito to DENV using whole mosquito, midgut, salivary glands at time points of 1–14 dpi, did not report any peroxiredoxins being differentially expressed due to DENV infection [16–20]. Except for a study that compared an *Ae. aegypti* dengue-susceptible (Cali-S) strain to a dengue-resistant (Cali-MIB) strain at 30 hpi, however, the peroxiredoxin-6 (prx-6) was exclusively expressed in the Cali-MIB strain, but was down-regulated upon DENV infection [39].

The bax inhibitor one is an evolutionarily conserved endoplasmic reticulum (ER) transmembrane protein that inhibits Bax induced apoptosis providing pro-survival properties against ER stress, reactive oxygen species accumulation, and metabolic imbalances [40, 41]. The subunit DPM3 was identified by a genome-wide CRISPR-Cas9 screen as part of the ER resident dolichol-phosphate mannose synthase (DPMS) complex functioning as host dependency factor for DENV infection and other flaviviruses [41]. Nevertheless, the experiment was exclusively conducted in a human HAP1 cell line, limiting direct correlation.

l(2)efl encodes a member of the small heat shock 20 (HSP20) protein family, which are induced in response to heat shock, cellular stress, pathogen infection, and function as chaperones to guide and stabilize misfolded proteins [42, 43]. A recent study in mosquito and CCL-125 cells derived from larvae of *Ae. aegypti* showed that the increased expression of l(2)efl decreased DENV-2 replication, whereas the suppression using gene-specific dsRNA resulted in enhanced DENV-2 replication [43]. Notably, silencing of multiple l(2)efl genes was required to successfully suppress DENV-2 replication and function [43].

Genes encoding cellular, retina development, mitochondrial electron transport functions, an antimicrobial peptide (holotricin-3), and several uncharacterized genes were significantly down-regulated due to DENV infection. Holotricin-3 is an antimicrobial peptide (AMPs) part of the mosquito innate immune effector response known to target and lyse bacterial membranes providing protection against invading microorganisms [44, 45].

Despite some DEGs only meeting the significance threshold of FC ≥2.0 at one or two of the time points, most DEGs were identified at 6 hpi, including genes functionally associated with methylation, DENV resistance, SUMOylation, endoribonuclease, classical and non-classical immune, and cellular stress response. These included the C5 cytosine methyltransferase (DNMT2), N6-adenosine-methyltransferase (m^6^A), peptidyl-prolyl cis-trans isomerase (AeFKBP1), ribonuclease H1, ribonuclease kappa, and SUMO genes, which were up-regulated due to DENV infection. DNMT2 encodes an RNA cytosine methyltransferase that functions as a m^5^C-writer on cellular and viral RNA substrates, and confers biological functions such as gene regulation, genome stability, and host defence [46–48]. m^6^A encodes a methyltransferase-like 3 that acts as a m^6^A-writer involved in RNA post-transcriptional modifications [49, 50]. Studies have shown that modifications such as m^5^C and m^6^A not only occur in RNA of eukaryotic cells but also in RNA of viruses, capable of regulating viral RNA functionality and viral replication [47, 49, 50]. A homolog of the human DENV resistance gene AeFKBP1B with anti-viral functions against DENV in *Ae. aegypti* cell line and *in vivo* was also among the up-regulated DEGs [51].

RNase H1 performs essential functions in biochemical processes associated with DNA replication, gene expression, DNA repair, and degradation of RNA, and is an endoribonuclease with the capability of degrading RNA/DNA hybrids generated during viral replication in a non-sequence-specific manner [52–54]. Further, studies have suggested that RNase H1 could play a vital role in anti-viral defence in eukaryotes and prokaryotes [55–57]. Ribonuclease kappa (RNASEK), is known to promote infection of acid-dependent viruses that rely on endocytosis and pH-dependent entry, including DENV [58, 59]. A study in *Aedes aegypti* Aa20 and Aag2 cells showed that RNASEK knockdown resulted in reduced DENV replication providing further evidence of its unique role in virus infection [59]. Furthermore, three essential genes (SUMO-activating enzyme E1, SUMO-conjugating enzyme UBC9, and E3 SUMO-protein ligase) of the SUMOylation pathway, known to play a key role in the regulation of host anti-viral defence against arbovirus infection such as Zika virus (ZIKV), Semliki Forest virus, and Bunyamwera virus in mosquito cells, were significantly up-regulated at 6 hpi [60].

Non-classical immune genes associated with pathogen recognition, such as cytochrome P450s (CYPs), leucine-rich repeat proteins (LRRs), and clip-domain serine proteases (CLIP-SPs) were present among all individual time points, being predominately up-regulated.

Cytochrome P450s (CYPs) are involved in cellular functions such as oxidative, cellular stress, immune response and detoxification of xenobiotic substances [61–63]. Previous studies in *Ae. aegypti* reported CYPs being differentially expressed in response to DENV, ZIKV, and Chikungunya virus (CHIKV) (Alphavirus) [16, 64–66].

LRR containing proteins are associated with pathogen recognition and innate immune response against viruses such as DENV, ZIKV and CHIKV in a variety of organisms including *Ae. aegypti* [65, 67–70]. Studies have found that *Ae. aegypti* responds to arbovirus infection by enhanced expression of immune-related genes such as LRRs, CLIP-SPs, and myeloid differentiation proteins that initiate *Ae. aegypti’s* immunity and function as Pattern Recognition Receptors (PRRs) [65–67, 70, 71]. These PRRs detect viral particles as Pathogen-Associated Molecular Patterns at the surface of the host cell [66, 71].

Additionally, a myeloid differentiation primary response protein 88 (MyD88) involved in the activation of the classical innate anti-viral mosquito immune response Toll pathway, was consistently up-regulated with a fold change difference of 2.21 and 2.23 at 6 hpi and 1 hpi, respectively [65, 66, 70, 72, 73]. Silencing of MyD88 in *Ae. aegypti* resulted in a significant increase in DENV titres [66, 73].

Further, the immune deficiency (IMD) gene encoding the core adaptor protein, shown to activate the IMD pathway upon arbovirus infection in *Ae. aegypti* and Aag2 cells [66, 74–76], was significantly up-regulated at 24 hpi. Also, AeFKBP1, previously identified as a DVR, and DNMT2 remained up-regulated at 2.79 and 2.17 folds, however, their expression was almost reduced by two-fold compared to 6 hpi, 4.05 and 4.67, respectively.

In addition to protein coding RNAs, a total of 258 lncRNAs were differentially expressed in Aag2.*w*AlbB cells in response to DENV infection, however, the majority (60.1%) were down-regulated in contrast to 39.9 % being up-regulated across the three time points combined (Table S2). lncRNAs are transcribed by eukaryotic cells and classified as regulatory RNAs that are longer than 200 nucleotides, however, do not encode any proteins. Nonetheless, like protein-coding RNAs, many lncRNA transcripts are also poly-adenylated and consequently detected in transcriptional analysis [77]. A variety of biological functions such as transcriptional and post-transcriptional modifications, differentiation of immune cells, and virus–host interactions have been associated with lncRNAs [77–80]. Studies in *Ae. aegypti* and *Ae. albopictus* found lncRNAs to be differentially expressed upon DENV-2 and ZIKV infections, with some implicated in the suppression of viral replication in mosquito cells [19, 81, 82]. Further, examining *Ae. aegypti* mosquitoes transinfected with *w*MelPop *Wolbachia* strain revealed that the transcript levels of several long intergenic non-coding RNAs significantly increased in *Wolbachia *-colonized mosquitoes [81].

Due to concentrating on early hours post-DENV infection, when viral inhibition occurs in the presence of *Wolbachia *, our results are not directly comparable to previous studies that mainly used samples from days (1-14) after infection. However, studies investigating the transcriptional response of *Ae. aegypti* mosquito and Aag2 cells to DENV infection at later stages between 1–14 dpi identified heat shock associated l(2)efl [20], and immune-related genes such as CYPs**,** CLIP-SPs, and LRRs [16, 17, 20, 83] among the up- and down-regulated DEGs. Despite these similarities, none of the studies above, detected peroxiredoxins, DNMT2 or m^6^A methylation, SUMOylation, Toll, and IMD-related genes. The only exception is Li *et al*. (2020) [20] which reported Toll and IMD signalling pathway genes as down-regulated due to DENV infection in Aag2 cells at 1, 2, 4 dpi. The genes involved in post-transcriptional modifications and detoxification identified in our study at early hours of DENV infection are reported here for the first time.

Our findings highlight that *Ae. aegypti* initiates the differential expression of immune-related genes involved in the recognition and suppression of virus at the early stages of DENV infection, which to our knowledge, have not been examined previously. Given the beneficial interactions between the arthropod host and *Wolbachia *, and the recently revealed biological importance of epigenetic regulation, further research is necessary to uncover their full contribution to anti-viral protection.

## Supplementary Data

Supplementary material 1

Supplementary material 2

Supplementary material 2
