# Supplementary material for: Transcriptional response of Wolbachia-transinfected Aedes aegypti mosquito cells to dengue virus at early stages of infection
Source: J Gen Virol. 2022 Jan 10;103(1):001694. doi: 10.1099/jgv.0.001694 (PMC8895618; doi:10.1099/jgv.0.001694)
Supplement: Supplementary material 1 [file jgv-103-1694-s001.pdf]

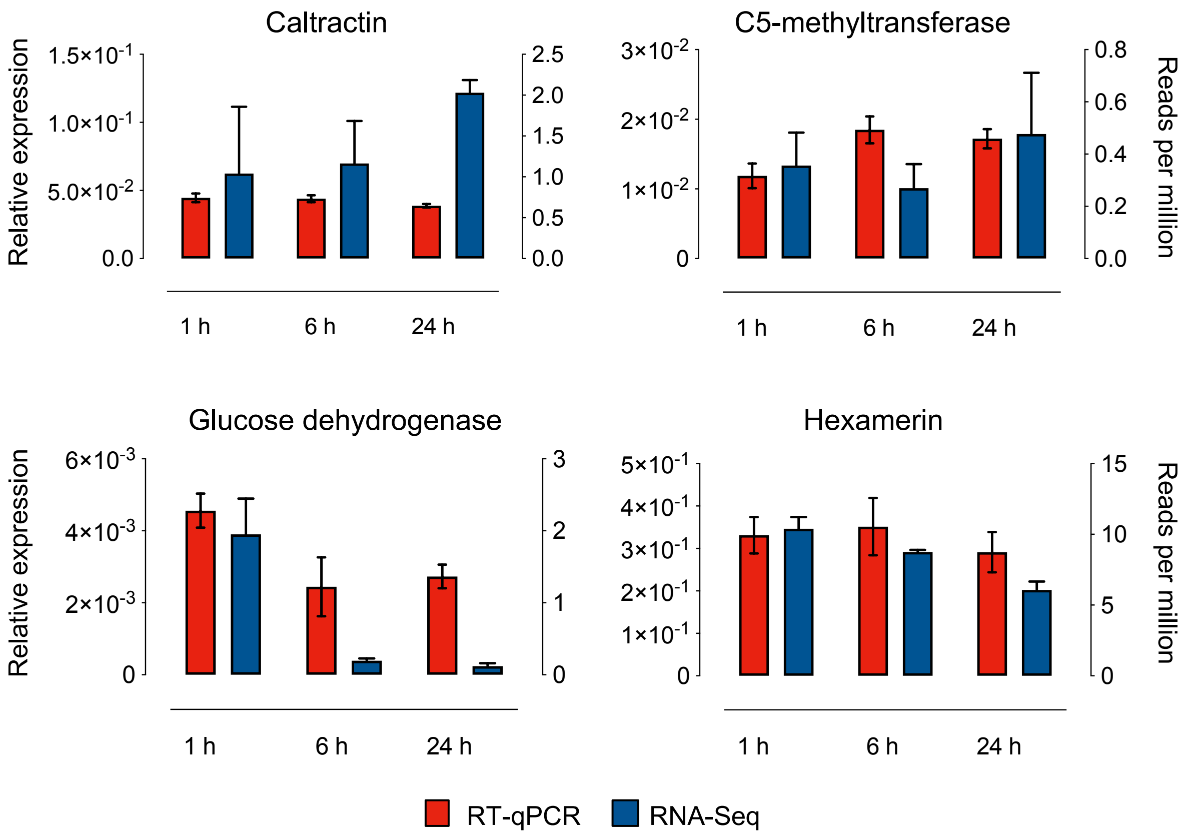

Figure S1: RT-qPCR validation of the differentially expressed genes (DEGs). The bar graphs represent the RNA-Seq normalized gene reads as counts per million and RT-qPCR relative expression results of the DEGs in uninfected *Wolbachia*-transinfected Aag2 cells at time points 1, 6, and 24 h post mock-infection. The error bars represent mean normalized expression (MNE) from the three biological replicates.

Table S3. Primer sequences used in this study.

Gene name	Primer Sequence (5'-3')
AeRPS17-qF	CACTCCGAGGTCCGTGGTAT
AeRPS17-qR	GGACACTTCGGGCACGTAGT
AeCALT-qF	GCGTTGCCAAAGAATTGGGT
AeCALT-qR	CTCCTGGTTGACTTCGCCAT
AeDNMT2-qF	GTAGAGCCCGAGTCCGAAAA
AeDNMT2-qR	ATTGGTAGAGTCTGGCGTGC
AeGDH-qF	TGCGACGACCTGAACACTTT
AeGDH-qR	AATCAGCAGGCAAGTCGGAA
AeHEX1-qF	AACGAGAACCGCGAGAACTT
AeHEX1-qR	GTCCTGCACGAACATCGAGA
AeL2EFL-qF	GCTCTCCGGCCATTGTATCA
AeL2EFL-qR	ACCGTCGAACGAACTGTCTC
AeRef2P-qF	AACCAGTAAGGACCGCGAAG
AeRef2P-qR	CTCCGAAGATTTGGCGCAAC
AeTFSOX8-qF	CAAACAGGAGCCCAAACTGC
AeTFSOX8-qR	CTTCTCCGCTTGCTCGATGA
AeVCES6-qF	CGTGGCTCCCTTCTTCGATT
AeVCES6-qR	TTAGCCAGGAATGGCCTTGG
